# Supplementary material for: Hybrid Models and Biological Model Reduction with PyDSTool
Source: PLoS Comput Biol. 2012 Aug 9;8(8):e1002628. doi: 10.1371/journal.pcbi.1002628 (PMC3415397; doi:10.1371/journal.pcbi.1002628)
Supplement: Text S4 — Complete source code for the PyDSTool package (version 0.88.120504). Includes API documentation and help files linking to web pages. This file is identical to the current public release on Sourceforge.net. (ZIP) [file pcbi.1002628.s004.zip › PyDSTool/html/PyDSTool.Toolbox.data_analysis-pysrc.html]

xml version="1.0" encoding="ascii"?


PyDSTool.Toolbox.data\_analysis


| Home | Trees | Indices | Help | PyDSTool |
| --- | --- | --- | --- | --- |

Package PyDSTool ::
Package Toolbox ::
Module data\_analysis

[hide private]

[frames] | no frames]

# Source Code for Module PyDSTool.Toolbox.data\_analysis

```
  1  """ 
  2  Data analysis utilities 
  3   
  4  Robert Clewley, August 2006 
  5  """ 
  6   
  7  from __future__ import division 
  8  from PyDSTool.Points import Point, Pointset 
  9  from PyDSTool.Interval import Interval 
 10  from PyDSTool.utils import intersect, filteredDict 
 11  from PyDSTool.errors import * 
 12  from PyDSTool.common import _num_types, _int_types, _seq_types, args, \ 
 13       sortedDictLists, sortedDictValues, sortedDictKeys 
 14  import numpy as npy 
 15  from numpy import array, dot, zeros, transpose, shape, extract, mean, std, \ 
 16       vstack, hstack, eye, ones, zeros, linalg, concatenate, \ 
 17       newaxis, r_, flipud, convolve, matrix, asarray 
 18  from numpy.linalg import norm 
 19  from scipy.signal import lfilter, butter 
 20  from scipy.optimize import minpack, optimize 
 21   
 22  try: 
 23      from mdp.nodes import PCANode 
 24  except: 


25 -    def PCANode(*args, **kwargs):


26          raise ImportError("MDP must be installed for this functionality")

 27  from matplotlib.pylab import figure, plot, hold, polyfit, \ 
 28       bar, subplot, axes, title, xlabel, ylabel, draw, semilogy 
 29  from copy import copy 
 30   
 31   
 32  _PCA_utils = ['doPCA', 'get_evec', 'pca_dim', 'get_residual', 
 33                'plot_PCA_residuals', 'plot_PCA_spectrum'] 
 34   
 35  _metric_utils = ['log_distances', 'log_distances_with_D'] 
 36   
 37  _analysis_utils = ['find_knees', 'whiten', 'find_closest_val', 'out_of_seq', 
 38                     'find_nearby_ball', 'find_nearby_annulus', 'data_bins', 
 39                     'find_central_point', 'find_diameter', 'find_from_sorted', 
 40                     'dist_between_datasets', 'find_recurrences'] 
 41   
 42  _fit_utils = ['get_linear_regression_residual', 'fitline'] 
 43   
 44  _filter_utils = ['lfilter_zi', 'filtfilt', 'lfilter', 'butter', 'rectify'] 
 45   
 46  __all__ = _PCA_utils + _metric_utils + _analysis_utils + _fit_utils + \ 
 47          _filter_utils 
 48   
 49  # ------------------------------------------------------------------------- 
 50   
 51  # Filter utils (reproduced from SciPy Cookbook) 
 52   


53 -def lfilter_zi(b,a):


54      #compute the zi state from the filter parameters. see [Gust96]. 
 55   
 56      #Based on: 
 57      # [Gust96] Fredrik Gustafsson, Determining the initial states in forward-backward 
 58      # filtering, IEEE Transactions on Signal Processing, pp. 988--992, April 1996, 
 59      # Volume 44, Issue 4 
 60   
 61      n=max(len(a),len(b)) 
 62   
 63      zin = (eye(n-1) - hstack( (-a[1:n,newaxis], 
 64                                   vstack((eye(n-2), zeros(n-2)))))) 
 65   
 66      zid=  b[1:n] - a[1:n]*b[0] 
 67   
 68      zi_matrix=linalg.inv(zin)*(matrix(zid).transpose()) 
 69      zi_return=[] 
 70   
 71      #convert the result into a regular array (not a matrix) 
 72      for i in range(len(zi_matrix)): 
 73          zi_return.append(float(zi_matrix[i][0])) 
 74   
 75      return array(zi_return)

 76   
 77   
 78   
 79   


80 -def filtfilt(b,a,x):


81      #For now only accepting 1d arrays 
 82      ntaps=max(len(a),len(b)) 
 83      edge=ntaps*3 
 84   
 85      if x.ndim != 1: 
 86          raise ValueError("Filiflit is only accepting 1 dimension arrays.") 
 87   
 88      #x must be bigger than edge 
 89      if x.size < edge: 
 90          raise ValueError, \ 
 91                "Input vector needs to be bigger than 3 * max(len(a),len(b)." 
 92   
 93      if len(a) < ntaps: 
 94          a=r_[a,zeros(len(b)-len(a))] 
 95   
 96      if len(b) < ntaps: 
 97          b=r_[b,zeros(len(a)-len(b))] 
 98   
 99      zi=lfilter_zi(b,a) 
100   
101      #Grow the signal to have edges for stabilizing 
102      #the filter with inverted replicas of the signal 
103      s=r_[2*x[0]-x[edge:1:-1],x,2*x[-1]-x[-1:-edge:-1]] 
104      #in the case of one go we only need one of the extremes 
105      # both are needed for filtfilt 
106   
107      (y,zf)=lfilter(b,a,s,-1,zi*s[0]) 
108   
109      (y,zf)=lfilter(b,a,flipud(y),-1,zi*y[-1]) 
110   
111      return flipud(y[edge-1:-edge+1])

112   
113   
114  # Principal Component Analysis utilities 
115   


116 -def doPCA(in_pts, D, tol=.8):


117      pts=copy(in_pts) 
118      pts.shape = (len(in_pts),D) 
119      p=PCANode(output_dim=tol) 
120      p.train(pts) 
121      p.stop_training() 
122      return p

123   
124   


125 -def get_evec(p, dim):


126      # extra call to array() to make it contiguous 
127      v = array(transpose(p.v)[dim-1]) 
128      v.shape = (totdim, 3) 
129      return v

130   
131   


132 -def pca_dim(pca, covering, data, refpt, tol=0.8):


133      if type(refpt) != int: 
134          refpt = refpt[0] 
135      pca[refpt] = doPCA(nhd(data, covering, refpt), tol) 
136      print "PCA eigenvalues for nhd #%i:"%refpt 
137      print pca[refpt].d 
138      return len(pca[refpt].d)

139   
140   


141 -def get_residual(pcanode, pts):


142      pvals = pcanode.execute(pts, pcanode.get_output_dim()) 
143      pproj = mat(pvals)*mat(transpose(pcanode.v)) 
144      res = pts-array(pproj) # orthogonal complement of projection onto PCs 
145      resval_part = norm(res) 
146      resval = norm(resval_part) 
147      return resval

148   
149   


150 -def plot_PCA_residuals(data, D=None, newfig=True, marker='o', 
151                         silent=False):


152      if D is None: 
153          D = shape(data)[1] 
154      p=doPCA(data, D, D) 
155      spec=zeros((D,1),float) 
156      for i in range(1,D): 
157          spec[i] = norm(p.d[:i]) 
158      res = transpose(sqrt(spec[-1]**2-spec**2)/spec[-1])[0] 
159      if not silent: 
160          print "L2-norm of PCA spectrum =", spec[-1] 
161          if newfig: 
162              figure() 
163              style='k'+marker 
164          else: 
165              style=marker 
166          semilogy(res,style) 
167      ##    title('PCA residuals') 
168          xlabel(r'$\rm{Dimension}$',fontsize=20) 
169          ylabel(r'$\rm{PCA \ residual}$',fontsize=20) 
170      return p, res

171   
172   


173 -def plot_PCA_spectrum(data, D):


174      p=doPCA(data, D, D) 
175      spec=zeros((D,1),float) 
176      for i in range(1,D): 
177          spec[i] = norm(p.d[:i]) 
178      figure();plot(spec,'ko') 
179      title('L2-norm of spectrum of singular values') 
180      xlabel('D') 
181      ylabel('|s|') 
182      return p

183   
184  # ----------------------------------------------------------------------------- 
185   
186   


187 -def log_distances(m, sampleix=0, doplot=True, quiet=True, logv=None, 
188                plotstyle=None, return_ixs=False):


189      """Log distances (L2-norm) of data points from a reference point (sampleix). 
190   
191      For multiple calls to this function on the same data, it's faster to 
192      pass logv precomputed. 
193   
194      return_ixs=True also returns the point indices from the sort to 
195      be returned in this argument in a third return value. 
196      """ 
197      npts = size(m,0) 
198      assert sampleix >= 0 
199      d = zeros((npts-1,), 'd') 
200      if rank(m) == 3: 
201          for i in xrange(npts): 
202              if sampleix != i: 
203                  try: 
204                      d[i] = norm(m[sampleix,:,:]-m[i,:,:]) 
205                  except IndexError: 
206                      # catch case when index is too large for npts-1 
207                      # so use the empty i=pix position (ordering doesn't matter) 
208                      d[sampleix] = norm(m[sampleix,:,:]-m[i,:,:]) 
209      elif rank(m) == 2: 
210          for i in xrange(npts): 
211              if sampleix != i: 
212                  try: 
213                      d[i] = norm(m[sampleix,:]-m[i,:]) 
214                  except IndexError: 
215                      # catch case when index is too large for npts-1 
216                      # so use the empty i=pix position (ordering doesn't matter) 
217                      d[sampleix] = norm(m[sampleix,:]-m[i,:]) 
218      else: 
219          raise ValueError("Rank of input data must be 2 or 3") 
220      if return_ixs: 
221          # return sorted indices in that list argument 
222          # (assumed to be an empty list) 
223          ixs = array(argsort(d)) 
224          d = d[ixs] 
225      else: 
226          # just sort 
227          d.sort() 
228      if not quiet: 
229          print "Chose reference point %i"%sampleix 
230          print "Min distance = %f, max distance = %f"%(d[0], d[-1]) 
231      logd = log(d).ravel() 
232      if logv is None: 
233          logv = log(range(1,len(d)+1)) 
234      nan_ixs = isfinite(logd)  # mask, not a list of indices 
235      logd = extract(nan_ixs, logd) 
236      logv = extract(nan_ixs, logv) 
237      if doplot: 
238          if plotstyle is None: 
239              plot(logd,logv) 
240          else: 
241              plot(logd,logv,plotstyle) 
242      if return_ixs: 
243          ixs = extract(nan_ixs, ixs) 
244          return (logv, logd, ixs) 
245      else: 
246          return (logv, logd)

247   
248   
249  # used for log_distances_with_D 
250  dict_tol = array([1e-8]) 
251   
252   


253 -def log_distances_with_D(m, sampleix, logv=None, return_ixs=False):


254      """Log distances (L2-norm) of data points from a reference point (sampleix). 
255      Additionally, it returns a list of the inter-point distances, 
256      relative to the reference point, and the inverse of this mapping. 
257   
258      For multiple calls to this function on the same data, it's faster to 
259      pass logv precomputed. 
260   
261      return_ixs=True returns the point indices from the sort in a fifth 
262      return value. This identifies the points in the returned 
263      list of inter-point distances and its inverse. 
264      """ 
265      npts = size(m,0) 
266      dk = zeros((npts-1,), 'd') 
267      d_inv = {} 
268      # do in two stages: ix 0->sampleix, sampleix+1->npts-1 
269      # but offset second stage indices in dk so there's a total of n-1 
270      # this is basically a slightly unrolled loop to make it a little faster 
271      if rank(m) == 3: 
272          for i in xrange(sampleix): 
273              dk[i] = norm(m[sampleix,:,:]-m[i,:,:]) 
274              if dk[i] in d_inv: 
275                  # ensure we find a unique distance for dictionary key 
276                  # (doesn't matter if we adjust it at very fine resolution 
277                  # to become unique) 
278                  done = 0 
279                  u = dk[i] 
280                  sgn = 1 
281                  lim=40 
282                  while done < lim: 
283                      u = dk[i] + sgn*done*dict_tol 
284                      if u[0] not in d_inv: 
285                          done = lim+1  # avoids exception below 
286                      else: 
287                          if sgn == -1: 
288                              sgn = 1 
289                          else: 
290                              done += 1 
291                              sgn = -1 
292                  if done == lim: 
293                      raise ValueError("Non-unique distance found") 
294                  dk[i] = u 
295                  d_inv[u[0]] = i 
296              else: 
297                  d_inv[dk[i]] = i 
298          for i in xrange(sampleix+1, npts): 
299              ki = i-1 
300              dk[ki] = norm(m[sampleix,:,:]-m[i,:,:]) 
301              if dk[ki] in d_inv: 
302                  # ensure we find a unique distance for dictionary key 
303                  # (doesn't matter if we adjust it at very fine resolution 
304                  # to become unique) 
305                  done = 0 
306                  u = dk[ki] 
307                  sgn = 1 
308                  lim=40 
309                  while done < lim: 
310                      u = dk[ki] + sgn*done*dict_tol 
311                      if u[0] not in d_inv: 
312                          done = lim+1  # avoids exception below 
313                      else: 
314                          if sgn == -1: 
315                              sgn = 1 
316                          else: 
317                              done += 1 
318                              sgn = -1 
319                  if done == lim: 
320                      raise ValueError("Non-unique distance found") 
321                  dk[ki] = u 
322                  d_inv[u[0]] = i 
323              else: 
324                  d_inv[dk[ki]] = i 
325      elif rank(m) == 2: 
326          for i in xrange(sampleix): 
327              dk[i] = norm(m[sampleix,:]-m[i,:]) 
328              if dk[i] in d_inv: 
329                  # ensure we find a unique distance for dictionary key 
330                  # (doesn't matter if we adjust it at very fine resolution 
331                  # to become unique) 
332                  done = 0 
333                  u = dk[i] 
334                  sgn = 1 
335                  lim=40 
336                  while done < lim: 
337                      u = dk[i] + sgn*done*dict_tol 
338                      if u[0] not in d_inv: 
339                          done = lim+1  # avoids exception below 
340                      else: 
341                          if sgn == -1: 
342                              sgn = 1 
343                          else: 
344                              done += 1 
345                              sgn = -1 
346                  if done == lim: 
347                      raise ValueError("Non-unique distance found") 
348                  dk[i] = u 
349                  d_inv[u[0]] = i 
350              else: 
351                  d_inv[dk[i]] = i 
352          for i in xrange(sampleix+1, npts): 
353              ki = i-1 
354              dk[ki] = norm(m[sampleix,:]-m[i,:]) 
355              if dk[ki] in d_inv: 
356                  # ensure we find a unique distance for dictionary key 
357                  # (doesn't matter if we adjust it at very fine resolution 
358                  # to become unique) 
359                  done = 0 
360                  u = dk[ki] 
361                  sgn = 1 
362                  lim=40 
363                  while done < lim: 
364                      u = dk[ki] + sgn*done*dict_tol 
365                      if u[0] not in d_inv: 
366                          done = lim+1  # avoids exception below 
367                      else: 
368                          if sgn == -1: 
369                              sgn = 1 
370                          else: 
371                              done += 1 
372                              sgn = -1 
373                  if done == lim: 
374                      raise ValueError("Non-unique distance found") 
375                  dk[ki] = u 
376                  d_inv[u[0]] = i 
377              else: 
378                  d_inv[dk[ki]] = i 
379   
380      else: 
381          raise ValueError("Rank of input data must be 2 or 3") 
382      d = copy(dk)  # dk remains unsorted 
383      if return_ixs: 
384          # return sorted indices in that list argument 
385          # (assumed to be an empty list) 
386          ixs = array(argsort(d)) 
387          d = d[ixs] 
388      else: 
389          # just sort 
390          d.sort() 
391      logd = log(d).ravel() 
392      if logv is None: 
393          logv = log(range(1,len(d)+1)) 
394      nan_ixs = isfinite(logd)  # mask, not a list of indices 
395      logd = extract(nan_ixs, logd) 
396      logv = extract(nan_ixs, logv) 
397      d = extract(nan_ixs, d) 
398  ##    for i in range(len(d)): 
399  ##        if nan_ixs[i] == 0: 
400  ##            try: 
401  ##                del d_inv[dk[i][0]] 
402  ##                print "Deleted key %i @ %f"%(i,dk[i][0]) 
403  ##            except KeyError: 
404  ##                print "Failed to delete key %i @ %f"%(i,dk[i][0]) 
405  ####                raise 
406      if return_ixs: 
407          ixs = extract(nan_ixs, ixs) 
408          return (logv, logd, d, d_inv, ixs) 
409      else: 
410          return (logv, logd, d, d_inv)

411   
412  # ---------------------------------------------------------------------------- 
413   


414 -def rectify(xa, full=False):


415      """Half-wave (default) or full-wave signal rectification: specify using 
416      optional full Boolean flag. 
417      xa must be an array""" 
418      if full: 
419          pos = npy.asarray(xa>0,int) 
420          return pos*xa - (1-pos)*xa 
421      else: 
422          return npy.asarray(xa>0,int)*xa

423   
424   
425   


426 -def get_linear_regression_residual(pfit, x, y, weight='', w=0):


427      """Also see scipy.stat.linregress for a more sophisticated version 
428      """ 
429      res = 0 
430      if weight == 'lo': 
431          for i in range(len(x)): 
432              res += ((y[i]-(pfit[0]*x[i]+pfit[1]))/(i+1)/w)**2 
433      elif weight == 'hi': 
434          l = len(x) 
435          for i in range(l): 
436              res += ((y[i]-(pfit[0]*x[i]+pfit[1]))/(l-i+1)/w)**2 
437      else: 
438          for i in range(len(x)): 
439              res += (y[i]-(pfit[0]*x[i]+pfit[1]))**2 
440      return sqrt(res)

441   
442   


443 -def fitline(x, y, lo=0, hi=1, doplot=True, quiet=True, linewidth=2):


444      """fitline takes the position of low and high fit limits and 
445      returns the slope. Integer indices in the x data set can be 
446      specified for the low and high limits, otherwise use a fraction 
447      between 0 and 1. 
448   
449      In application to fractal dimension estimation, if 
450      x = log d (radius, a.k.a. inter-point distance) and 
451      y = log v (index, a.k.a. estimate of volume at a given radius) 
452      then the slope is the dimension estimate of the data set. 
453   
454      Also see scipy.stat.linregress for a more sophisticated version 
455      """ 
456      lendat = len(x) 
457      assert hi > lo 
458      if lo >= 0 and hi <= 1: 
459          loix = int(lendat*lo) 
460          hiix = int(lendat*hi) 
461      elif lo >=0 and hi > 0: 
462          loix = int(lo) 
463          hiix = int(hi) 
464      else: 
465          raise ValueError("Invalid low and high fit limits") 
466      if not quiet: 
467          print "lo ix %d, hi ix %d, max ix %d"%(loix, hiix, lendat-1) 
468      pfit = polyfit(x[loix:hiix],y[loix:hiix],1) 
469  #    print pfit 
470      if doplot: 
471          plot([x[loix],x[hiix]], 
472               [x[loix]*pfit[0]+pfit[1],x[hiix]*pfit[0]+pfit[1]], 
473               linewidth=linewidth) 
474      return pfit[0]

475   
476   
477   
478  # ---------------------------------------------------------------------------- 
479   
480  # Data analysis utilities 
481   


482 -class data_bins(object):


483      """Class for data binning. Indexed by bin number 0 - (num_bins-1). 
484   
485      Initialization arguments: 
486              coordinate -- name of coordinate that occupies bins 
487              bin_ords   -- ordered sequence of n bin ordinates (integers or floats) 
488                            defining n-1 bins 
489              valuedict  -- optional dictionary containing initial values for 
490                            bins: keys = ordinate values, 
491                                  values = coordinate values 
492              tolerance  -- for resolving bin edges with finite precision 
493                            arithmetic (defaults to 1e-12) 
494      """ 
495   


496 -    def __init__(self, coordinate, bin_ords, valuedict=None, tolerance=1e-12):


497          assert isincreasing(bin_ords), \ 
498                  "Bin ordinates must be given as a sequence in increasing order" 
499          # bins are referenced internally by integer index 
500          self.num_bins = len(bin_ords)-1 
501          assert self.num_bins > 0, "Must be more than 2 ordinates to define bins" 
502          self.bin_ords = array(bin_ords) 
503          self.bins = dict(zip(range(self.num_bins),[0]*self.num_bins)) 
504          self.coordinate = coordinate 
505          self.tolerance = tolerance 
506          self.intervals = [Interval('bin_%s'%i, float, 
507                                  (bin_ords[i],bin_ords[i+1]), \ 
508                                  abseps=tolerance) for i in range(self.num_bins)] 
509          self.midpoints = (self.bin_ords[1:]+self.bin_ords[:-1])/2. 
510          if valuedict is not None: 
511              for k, v in valuedict.iteritems(): 
512                  self[self.resolve_bin_index(k)] = v

513   


514 -    def resolve_bin_index(self, ordinate):


515          """Find bin number (index) associated with ordinate""" 
516          try: 
517              # return first bin index for which ordinate in bin interval is 
518              # 'uncertain' or 'contained' 
519              return [interval.contains(ordinate) is not notcontained \ 
520                         for interval in self.intervals].index(True) 
521          except ValueError: 
522              # no bin found 
523              raise ValueError("No corresponding bin for this ordinate")

524   


525 -    def __call__(self, ordinate):


526          """Return content of the bin that ordinate resolves to. 
527          """ 
528          return self.bins[self.resolve_bin_index(ordinate)]

529   


530 -    def __getitem__(self, bin_index):


531          if isinstance(bin_index, _int_types): 
532              # bin_index is a single index, so... 
533              # return first bin for which ordinate in bin interval is 
534              # 'uncertain' or 'contained' 
535              return self.bins[bin_index] 
536          elif isinstance(bin_index, slice): 
537              if bin_index.step is not None: 
538                  if bin_index.step != 1: 
539                      raise ValueError("Cannot step in slice of bins") 
540              if bin_index.start is None: 
541                  start = 0 
542              else: 
543                  start = bin_index.start 
544              if bin_index.stop is None: 
545                  stop = self.num_bins 
546              else: 
547                  stop = bin_index.stop 
548              assert start < stop, \ 
549                         "Slice must go in increasing order" 
550              new_bin_ords = self.bin_ords[start:stop+1] 
551              b = bin(self.coordinate, new_bin_ords, 
552                      tolerance=self.tolerance) 
553              for x in new_bin_ords[:-1]: 
554                  bin_ix = self.resolve_bin_index(x+self.tolerance) 
555                  b.bins[bin_ix] = self.bins[bin_ix] 
556              return b 
557          else: 
558              raise ValueError("Invalid indexing of bins")

559   


560 -    def __setitem__(self, bin_index, value):


561          """Set value of a given bin number (index).""" 
562          self.bins[bin_index] = value

563   


564 -    def increment(self, ordinate, inc=1):


565          """Increment the bin corresponding to the ordinate argument. 
566          Optional argument inc sets increment amount (default 1). 
567          """ 
568          self.bins[self.resolve_bin_index(ordinate)] += inc

569   


570 -    def bin(self, values):


571          """Increment bin contents according to values passed.""" 
572          if isinstance(values, _seq_types): 
573              for v in values: 
574                  self.increment(v) 
575          else: 
576              self.increment(values)

577   


578 -    def clear(self):


579          """Reset all bins to have zero contents.""" 
580          self.bins = dict(zip(range(self.num_bins),[0]*self.num_bins))

581   


582 -    def __str__(self):


583          return "Binned data with intervals %s:\n By bin number: %s"%(str([i.get() \ 
584                       for i in self.intervals]), str(self.bins))

585   
586      __repr__ = __str__ 
587   


588 -    def to_pointset(self):


589          """Convert to a pointset""" 
590          bin_ixs = xrange(self.num_bins) 
591          return Pointset(indepvararray=range(self.num_bins), 
592                          coorddict={self.coordinate: [self.bins[ix] \ 
593                                                          for ix in bin_ixs], 
594                                     'bin_limit_lo': [self.intervals[ix][0] \ 
595                                                          for ix in bin_ixs], 
596                                     'bin_limit_hi': [self.intervals[ix][1] \ 
597                                                          for ix in bin_ixs] 
598                                      })

599   


600 -    def to_arrays(self):


601          """Convert to two arrays of bin indices and values""" 
602          x, y = sortedDictLists(self.bins, byvalue=False) 
603          return (array(x), array(y))

604   


605 -    def values(self):


606          return sortedDictValues(self.bins)

607   


608 -    def keys(self):


609          return sortedDictKeys(self.bins)

610   


611 -    def mean(self):


612          """Mean of binned data""" 
613          a = self.midpoints*array(sortedDictValues(self.bins)) 
614          return sum(a) / sum(self.bins.values())

615   


616 -    def std(self):


617          """Standard deviation of binned data""" 
618          vals = array(sortedDictValues(self.bins)) 
619          a = self.midpoints*vals 
620          sum_bin_vals = sum(self.bins.values()) 
621          mean_val = sum(a) / sum_bin_vals 
622          return sqrt(sum(((self.midpoints-mean_val)*vals)**2) / sum_bin_vals)

623   
624   


625 -def find_closest_val(x, a, eps=1e-7, which_norm=2):


626      """Find one value in a closer than eps to x""" 
627      for aval in a: 
628          if norm(x-aval, which_norm) < eps: 
629              return aval 
630      return None

631   
632   


633 -def find_nearby_ball(data, refix, r, which_norm=2, include_ref=False):


634      """Return indices of all points in data that are inside a ball of 
635      radius r from the reference point (not included).""" 
636      nearby = [] 
637      refpt = data[refix] 
638      if include_ref: 
639          for i in xrange(size(data,0)): 
640              if norm(refpt-data[i], which_norm) <= r: 
641                  nearby.append(i) 
642      else: 
643          for i in xrange(size(data,0)): 
644              if i != refix: 
645                  if norm(refpt-data[i], which_norm) <= r: 
646                      nearby.append(i) 
647      return nearby

648   


649 -def find_nearby_annulus(data, refix, rlo, rhi, which_norm=2):


650      """Return a list containing input data's indices of all neighbours of the 
651      reference point within the range of distances d such that dlo < d < dhi 
652      """ 
653      assert rlo > 0 and rhi > rlo 
654      nearby = [] 
655      for i in xrange(size(data,0)): 
656          if i != refix: 
657              d = norm(data[refix]-data[i], which_norm) 
658              if d > rlo and d < rhi: 
659                  nearby.append(i) 
660      return nearby

661   


662 -def find_central_point(data, which_norm=2):


663      """Find the index of a point closest to the 'centre' of a data set. 
664      The centre is defined to be where the mean of the points' position 
665      vectors relative to the centre is minimized in each direction.""" 
666      dim = data.shape[1] 
667      centre = mean(data,0) 
668      ds = array([norm(p, which_norm) for p in data-centre]) 
669      ds_sort_ixs = ds.argsort() 
670      return ds_sort_ixs[0]

671   
672   


673 -def find_recurrences(data, refix, r, times=None, ignore_small=0, 
674                       which_norm=2):


675      """Find recurrences of a trajectory in a ball of radius r centred at the 
676      reference point given by refix in data. 
677   
678      If recurrence times are desired, pass an associated time array corresponding 
679      to the points in the data. 
680   
681      If ignore_small > 0, recurrences of fewer than this number of data points 
682         will be treated as anomalous and ignored completely. 
683   
684      Returns a structure (class common.args) having attributes: 
685          partitions -- pairs of entry and exit indices for each recurrence in 
686                  the ball 
687          ball_ixs -- all consecutive indices into data for each recurrence 
688          partitions_lengths -- number of indices in each partition 
689          rec_times -- times to next recurrence in the set from previous 
690      """ 
691      if times is not None: 
692          assert len(times) == len(data), "Times must have same length as data" 
693      # ball_ixs is already sorted 
694      ball_ixs = find_nearby_ball(data, refix, r, which_norm, include_ref=True) 
695      result = args(ball_ixs=[], partitions=[], partition_lengths=[], 
696                    rec_times=None) 
697      if len(ball_ixs) == 0: 
698          # No points found in ball 
699          return result 
700      # find boundaries of each partition between contiguous indices 
701      partition_bds = out_of_seq(ball_ixs) 
702      if len(partition_bds) == 0: 
703          result.ball_ixs = ball_ixs 
704          result.partitions = [(ball_ixs[0], ball_ixs[-1])] 
705          result.partition_lengths = [ball_ixs[-1] - ball_ixs[0] + 1] 
706          if times is not None: 
707              # only update this if times present to be consistent with other 
708              # changes of rec_times from default None 
709              result.rec_times = [Inf] 
710          return result 
711      else: 
712          plen = ball_ixs[partition_bds[0]-1] - ball_ixs[0] + 1 
713          if plen > ignore_small: 
714              partitions = [(ball_ixs[0], ball_ixs[partition_bds[0]-1])] 
715          else: 
716              partitions = [] 
717      # find contiguous indices (and corresponding times) 
718      for i in range(len(partition_bds)-1): 
719          plo = ball_ixs[partition_bds[i]] 
720          phi = ball_ixs[partition_bds[i+1]-1] 
721          plen = phi - plo + 1 
722          if plen > ignore_small: 
723              partitions.append((plo, phi)) 
724      plo = ball_ixs[partition_bds[-1]] 
725      phi = ball_ixs[-1] 
726      plen = phi - plo + 1 
727      if plen > ignore_small: 
728          partitions.append((plo, phi)) 
729      result.ball_ixs = ball_ixs 
730      result.partitions = partitions 
731      result.partition_lengths = [p[1]-p[0]+1 for p in partitions] 
732      if times is None: 
733          return result 
734      else: 
735          result.rec_times = recurrence_times(times, partitions) 
736          return result

737   
738   


739 -def find_diameter(data, eps, which_norm=2):


740      """Find approximate diameter of data set, up to tolerance defined by eps > 0. 
741   
742      Data assumed to be a rank-2 array. 
743      """ 
744      assert eps > 0, "Tolerance must be positive" 
745      num_pts = len(data) 
746      if num_pts > 400: 
747          # If data well distributed, assume that 100 sample points is a good 
748          # initial set to test 
749          sample_rate = int(ceil(num_pts/100.)) 
750      else: 
751          # small enough data set to do by brute force on all points 
752          sample_rate = 1 
753      old_diam = -Inf 
754      delta = Inf  # change in estimate of radius on each iteration 
755      while delta > eps: 
756          #print "Sample rate = ", sample_rate 
757          downsampled = data[::sample_rate] 
758          mind, diam = dist_between_datasets(downsampled, downsampled, 
759                                               which_norm) 
760          if sample_rate == 1: 
761              # can go no further! 
762              delta = 0 
763              break 
764          else: 
765              sample_rate = int(ceil(sample_rate/2.)) 
766          delta = diam - old_diam 
767          old_diam = diam 
768          #print old_diam, delta 
769      return diam

770   
771   


772 -def dist_between_datasets(data1, data2, which_norm=2):


773      maxd = -Inf 
774      mind = Inf 
775      for v1 in data1: 
776          for v2 in data2: 
777              d = norm(v1-v2, which_norm) 
778              if d > maxd: 
779                  maxd = d 
780              elif d < mind: 
781                  mind = d 
782      return (mind, maxd)

783   
784   


785 -def out_of_seq(a, inc_only=False):


786      """Determine if and where an integer array a is not increasing or 
787      decreasing in sequence by 1. 
788      """ 
789      v_old = a[0] 
790      i = 0 
791      out = [] 
792      dirn = 0 
793      for v in a[1:]: 
794          i +=1 
795          if v == v_old+1: 
796              if dirn == -1: 
797                  out.append(i) 
798              dirn = 1 
799          elif v == v_old-1: 
800              if dirn == 1: 
801                  out.append(i) 
802              dirn = -1 
803          else: 
804              dirn = 0 
805              out.append(i) 
806          v_old = v 
807      return out

808   
809   


810 -def whiten(data):


811      """Subtract mean and divide by standard deviation in each column of data""" 
812      wdata=zeros(shape(data),Float) 
813      for d in range(shape(data)[1]): 
814          x = data[:,d] 
815          wdata[:,d] = (x-mean(x))/std(x) 
816      return wdata

817   
818   


819 -def find_knees(data, tol=1., inlog=False, verbose=False):


820      """ 
821      Find one or more 'knees' in data, according to high second derivatives. 
822   
823      inlog option finds knees in the logs of the data entries. 
824      tol=1. works well if inlog=False 
825      tol=0.3 works well if inlog=True 
826      """ 
827      knee_ixs = [] 
828      for i in range(1,len(data)-1): 
829          if inlog: 
830              frac = log(data[i+1])+log(data[i-1])-2*log(data[i]) 
831          else: 
832              d2 = data[i+1]+data[i-1]-2*data[i] 
833              try: 
834                  frac = d2/data[i] 
835              except ZeroDivisionError: 
836                  frac = Inf 
837          if verbose: 
838              print i, data[i], frac 
839          if frac > tol and frac < Inf: 
840              knee_ixs.append((i,frac)) 
841      if verbose: 
842          print "High second derivatives at: ", knee_ixs, "\n" 
843      knees = [] 
844      found = False  # in a contiguous segment of high second derivatives 
845      curr_kixs = [] 
846      old_kix = None 
847      for i in range(len(knee_ixs)): 
848          process = False 
849          kix, frac = knee_ixs[i] 
850          if verbose: 
851              print "Processing knee at index", kix 
852          if kix-1 == old_kix: 
853              if i == len(knee_ixs)-1: 
854                  # this is the last index so have to process 
855                  process = True 
856              if found: 
857                  curr_kixs.append(i) 
858              else: 
859                  curr_kixs = [i-1, i] 
860                  found = True 
861          else: 
862              process = old_kix is not None 
863          if verbose: 
864              print old_kix, found, curr_kixs 
865          if process: 
866              if found: 
867                  found = False 
868                  if verbose: 
869                      print "Current knee indices:", curr_kixs, 
870                      print [knee_ixs[k] for k in curr_kixs] 
871                  all_d2 = array([knee_ixs[k][1] for k in curr_kixs]) 
872                  ixs_sort = argsort(all_d2) 
873                  max_ix = ixs_sort[-1] 
874                  knees.append(knee_ixs[curr_kixs[max_ix]][0]) 
875                  curr_kixs = [] 
876              else: 
877                  if verbose: 
878                      print "Appending knee index", old_kix 
879                  knees.append(old_kix) 
880          old_kix = kix 
881      # add final singleton index of high derivative, if any 
882      if not found and old_kix not in knees: 
883          if verbose: 
884              print "Appending knee index", old_kix 
885          knees.append(old_kix) 
886      return knees

887   
888   
889  # ----------------------------------------------------------------------------- 
890   
891  # Internal helper functions 
892   


893 -def recurrence_times(ts, partitions):


894      """Internal function for use by find_recurrences""" 
895      rtimes = [] 
896      if len(partitions) == 1: 
897          return [Inf] 
898      for i in range(len(partitions)-1): 
899          (plo, phi) = partitions[i] 
900          rtimes.append(ts[partitions[i+1][0]]-ts[partitions[i][1]]) 
901      return rtimes

902   
903   


904 -def find_from_sorted(x, v, next_largest=1, indices=None):


905      if isinstance(v, _seq_types): 
906          # sequence v 
907          return [find(array(x), y, next_largest, indices) for y in v] 
908      else: 
909          # singleton v 
910          return find(array(x), v, next_largest, indices)

911   


912 -def colormap(mag, cmin, cmax):


913      """ 
914      Return a tuple of floats between 0 and 1 for the red, green and 
915      blue amplitudes. 
916      Function originally named floatRgb and written by Alexander Pletzer, 
917      from the Python Cookbook. 
918      """ 
919   
920      try: 
921          # normalize to [0,1] 
922          x = float(mag-cmin)/float(cmax-cmin) 
923      except: 
924          # cmax = cmin 
925          x = 0.5 
926      blue = min((max((4*(0.75-x), 0.)), 1.)) 
927      red  = min((max((4*(x-0.25), 0.)), 1.)) 
928      green= min((max((4*math.fabs(x-0.5)-1., 0.)), 1.)) 
929      return (red, green, blue)

930   
931   


932 -def dump_progress(x, tot, filename='progress_info.dat'):


933      """Send progress report to a file""" 
934      f=open(filename, 'w') 
935      f.write("Progress: %d / %d \n"%(x,tot)) 
936      f.write("Date and time of update: " + " ".join([str(x) for x in localtime()[:-3]]) + "\n\n") 
937      f.close()

938   
939   


940 -def second_diff(data, i):


941      """Naive second difference formula at position i of data""" 
942      return data[i+1]+data[i-1]-2*data[i]

943   
944   


945 -def _adjust_ix(i, n):


946      """Internal helper function""" 
947      if i >= n: 
948          return i+1 
949      else: 
950          return i

951
```

  


| Home | Trees | Indices | Help | PyDSTool |
| --- | --- | --- | --- | --- |

|  |  |
| --- | --- |
| Generated by Epydoc 3.0.1 on Fri May 4 15:24:23 2012 | http://epydoc.sourceforge.net |
